# Antimicrobial Peptides and Nanotechnology, Recent Advances and Challenges

**DOI:** 10.3389/fmicb.2018.00855

**Published:** 2018-05-08

**Authors:** Lubhandwa S. Biswaro, Mauricio G. da Costa Sousa, Taia M. B. Rezende, Simoni C. Dias, Octavio L. Franco

**Affiliations:** ^1^Center of Proteomic and Biochemical Analysis, Genomic Sciences and Biotechnology Program, Catholic University of Brasília, Brasília, Brazil; ^2^Catholic University of Brasília, Brasília, Brazil; ^3^Health Science Program, University of Brasília, Brasília, Brazil; ^4^S-Inova Biotech, Biotechnology Program, Dom Bosco Catholic University, Campo Grande, Brazil

**Keywords:** antimicrobial peptides, drug delivery systems, nanotechnology, resistance, novel therapy

## Abstract

Antimicrobial peptides are sequences of amino acids, which present activity against microorganisms. These peptides were discovered over 70 years ago, and are abundant in nature from soil bacteria, insects, amphibians to mammals and plants. They vary in amino acids number, the distance between amino acids within individual peptide structure, net charge, solubility and other physical chemical properties as well as differ in mechanism of action. These peptides may provide an alternative treatment to conventional antibiotics, which encounter resistance such as the peptide nisin applied in treating methicillin resistant *Staphylococcus aureus* (MRSA) or may behave synergistically with known antibiotics against parasites for instance, nisin Z when used in synergy with ampicillin reported better activity against *Pseudomonas fluorescens* than when the antibiotic was alone. AMPs are known to be active against viruses, bacteria, fungi and protozoans. Nanotechnology is an arena which explores the synthesis, characterization and application of an array of delivery systems at a one billionth of meter scale. Such systems are implemented to deliver drugs, proteins, vaccines, and peptides. The role of nanotechnology in delivering AMPs is still at its early development stage. There are challenges of incorporating AMPs into drug delivery system. This review intends to explore in depth, the role of nanotechnology in delivering AMPs as well as presenting the current advances and accompanying challenges of the technology.

## Introduction to Antimicrobial Peptides and Their Importance

Antimicrobial peptides (AMPs) can be considered as natural antibiotics produced by animals, plants, protozoa, fungi, and bacteria (Lopez-Abarrategui et al., 2012). AMPs contain 5–50 amino acid chains and are generally composed of L-amino acids defined in secondary structures formed by α-helices, β-sheets or both (Sirtori et al., 2008). This type of biomolecule may have an amphipathic or cationic structure and may exhibit a broad spectrum of action, against Gram-positive and negative bacteria, fungi, viruses and protozoa (Zhao et al., 2013). In addition, AMPs may present other activities such as defense mechanism, antitumor, or even regenerative (Conlon et al., 2014; Roudi et al., 2017). For these reasons, their biotechnological potential has been studied in all of these fields (Conlon et al., 2014), making new pharmacological active principles possible in many health areas (Roudi et al., 2017).

The mechanism of action of AMPs has not yet been fully elucidated. Accepted models have reported intracellular and extracellular interference (Scocchi et al., 2016). The interaction of peptides with phospholipid membranes of microorganisms has been reported, mainly due to their biochemical properties. Based on the amino acid sequence, innate features, such as amphipathic charge, structure and hydrophobicity, have been defined through a local thinning layer by expansion of the outer membrane (Scocchi et al., 2016). In addition, other studies discuss the possibility of channels, pores or cracks forming in the membrane (Matsuzaki, 1999). This mechanism of action has been called a barrel steve, and it allows extravasation through interaction with selective ion channels (Gkeka and Sarkisov, 2010). AMPs can also act in a ‘carpet’ form, where they penetrate the phospholipid membrane, forming a carpet and promoting the membrane’s destabilization (Lee et al., 2010). There are some studies that suggest the mechanism of toroidal pore formation, where the peptides are associated with propellers of phospholipid membranes (Tamba and Yamazaki, 2005). Moreover, in the mechanism of action known as detergent, peptides can cause rupture of the layers and consequent loss of ions (Wimley, 2010). Some studies consider the action of AMPs on enzymes, gene transcription and DNA synthesis (Wang et al., 2014). To date, there have been over 5000 AMPs reported (Bahar and Ren, 2013), with different ways of acting.

At the same time as AMPs emerge as a new antimicrobial therapeutic, resistance to available antibiotics has become a worldwide problem (Kaczor et al., 2017). Thus, as favorable possibilities, the use of AMPs has been studied not only against susceptible pathogens, but also against persistent bacteria and fungi (Jiang et al., 2014; Stedt et al., 2014).

In this context, since AMPs have a wide spectrum of activity and do not interact with specific receptors, it is rare to observe resistance phenotypes (Sun et al., 2018). However, some cases have already been documented, such as the resistance to dermicidin developed by *Staphylococcus aureus*, and the reduction of sensitivity to cationic AMPs developed by some bacteria (Ageitos et al., 2017). Several structural studies suggest that the dynamics and/or conformation of the peptides themselves can significantly affect their mechanism of action (Bahar and Ren, 2013; Kang et al., 2017).

Isolating natural AMPs is challenged by their low yield and undesired impurities, and so synthetic peptides are becoming more popular in scientific studies (Diehnelt, 2013). Peptide design allows amino acid residue modifications, increasing their antimicrobial and immunomodulatory capacity, or even improving their resistance against degradation (Riedl et al., 2011). Synthetic peptides can also be expressed in other organisms through heterologous expression techniques, allowing a reduction in price when produced on a large scale (Wang et al., 2014).

Although many AMPs show high antibacterial activity, some have undesirable characteristics for clinical use. Among these reasons are: (i) toxicity to eukaryotic cells, which can lead to hemolysis, nephrotoxicity and neurotoxicity; (ii) susceptibility to proteolysis by bacterial proteases; and (iii) the pharmacokinetic profile of AMPs is not well understood, and only a few clinical studies have been developed with these biomolecules (Navon-Venezia et al., 2002; Schmidtchen et al., 2002; Falagas and Kasiakou, 2005; Craik et al., 2013; Grassi et al., 2017). However, extensive studies have been carried out to improve these aspects (Maloy and Kari, 1995; Chou et al., 2008). Also, these peptides are expensive to synthesize on a mass scale in comparison to conventional antibiotics (Otvos and Wade, 2014).

The advances in nanobiotechnology have allowed different nanostructures to work as a good strategy to minimize the undesirable characteristics of natural and synthetic AMPs (Umerska et al., 2017). It has been reported that peptides in nanostructures presented lower cytotoxicity, reduced degradation and increased efficiency at the desired target (Zelezetsky and Tossi, 2006; Sun et al., 2017; Wang et al., 2017). Therefore, nanostructures can contribute to the production of these biomolecules in industry and their implementation on the market. Nano-carriers are drug delivery systems that present a number of advantages, such as the protection of the desired peptides, vaccines and drugs against extracellular degradation, as well as target treatment selectivity and drug pharmacokinetic profile improvement (Sadat et al., 2016). Over the last five decades, several types of drug delivery systems have been explored to encapsulate drugs and other biomolecules in general (Torchilin, 2006). These include liposomes (Bangham et al., 1965; Lopez-Berestein et al., 1985; Lasic, 1998; Torchilin, 2007; Haley and Frenkel, 2008; Steichen et al., 2013), micelles (Lavasanifar et al., 2002; Moghimi et al., 2005; Mu et al., 2005; Liechty and Peppas, 2012) dendrimers (Hughes, 2005; Majoros et al., 2006; Kurtoglu et al., 2010; Rai et al., 2010; Arpicco et al., 2015) and polymeric nanoparticles (Czerniawska, 1970; Jain, 2000; Brigger et al., 2002; Panyam et al., 2003; Ratner et al., 2004; Reis et al., 2006a; Guterres et al., 2007; Stevanović et al., 2007; Faraji and Wipf, 2009; Muthu and Singh, 2009; Kumari et al., 2010; Rao and Geckeler, 2011; Chen and Liu, 2012). In this review, the role of various drug delivery systems applied in encapsulating AMPs will be discussed.

## Nanotechnology Involvement in the Delivery of Antimicrobial Peptides

**FIGURE 1:**
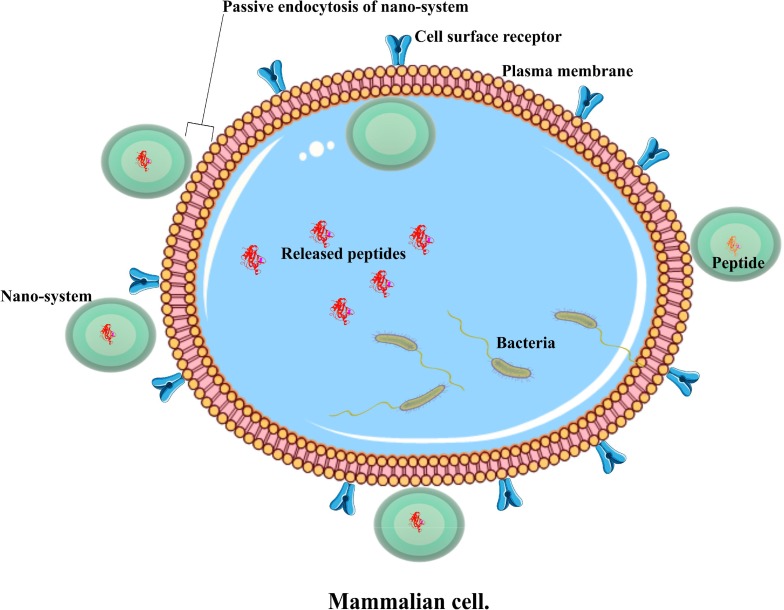
An illustration of a passive nano system, demonstrating antimicrobial peptide delivery into an infected cell.

Nanotechnology employs two main approaches in encapsulating AMPs. The non-directed one is known as passive delivery and involves conventional nano delivery systems which do not possess surface modification of their structure to guide the nano-carrier; this can be manipulated through controlling the nano-carrier’s size and shape (Storma et al., 1995) as represented in **Figure 1**. Directed delivery is referred to as active targeting and involves modification of the surface chemistry of the nano-carrier with ligands or other moieties to allow interaction of the nano-carrier and the intended site (Lamprecht et al., 2001). However, developing viable drug delivery systems for clinical trial still remains a challenge (Turos et al., 2007). Therefore, these two approaches of nano delivery systems will be discussed.

Both delivery systems have a number of pros and cons. Passive systems tend to possess fewer agents in their composition compared to active ones. As the former have fewer agents in their composition, this makes it relatively easier to prepare them. Additionally, a passive system involves encapsulating peptides without providing an extra surface modification, while an active system contains modified surface-carrying ligands, or other moieties to facilitate its interaction with infected cells and increases the overall drug transport at such specific sites (Solaro et al., 2010; Hunter et al., 2012). The terms nano-carriers and nano delivery system will be used interchangeably in this review.

### Antimicrobial Peptides in Passive Nano Delivery Systems

An evaluation will be provided of chosen AMPs that are being studied as potential antimicrobials, whether *in vitro*, *in vivo* or both, and a few clinical trials reported for such peptides. Additionally, other potential applications of peptides in the food industry will be examined. Some of the cases are presented in **Table 1**.

**Table 1:** An overview of antimicrobial peptides contained/adsorbed in various passive nano delivery systems and their various biological applications.

Encapsulant	Nano delivery system	Antimicrobial target	Application	Reference
Cyclosporin	Amorpohus nanoparticles		Penetration study pig skin model	Romero et al., 2016
	Chitosan nanoparticles		Biodistribution	De Campos et al., 2004
	Polylactic-co-glycolide-co-caprolactone nanoparticles		Clinical trial for high risk keratoplasty	Shi et al., 2013
Nisin	Liposomes	*Lactococcus lactis*	Milk fermentation	Laridi et al. (2003)
		*Listeria monocytogenes*		
	Solid lipid nanoparticles	*Lactobacillus plantarum* and *Candida albicans*	Cheese production	Prombutara et al., 2012
	Polycaprolactone nanoparticles	*Staphylococcus aureus*	Antifungal therapeutic	Coli et al., 2016
	Polyvinyl alcohol nanofibers			Wang et al., 2015
Vancomycin	PLGA nanoparticles	*Staphylococcus*		Yousry et al., 2017
	Polycaprolactone microparticles	Gram-positive bacteria	Bone transplantation	Looss et al., 2001
	PLGA microparticles		Biodistribution	Gavini et al., 2004
	PLGA nanofibers		Post operative Central Nervous system infection	Tseng et al., 2015
	Polycaprolactones microparticles	*Pseudomonas aeruginosa*	Bone implantation	Le Ray et al., 2003
	Solid lipid nanoparticles			Severino et al., 2017
	Silver nanoparticles	*Pseudomonas aeruginosa*		Lambadi et al., 2015
Polymyxin	Solid lipid nanoparticles	*Pseudomonas aeruginosa*		Severino et al., 2017
	Liposomes		Permeation	Alipour et al., 2008
Synthetic peptides (FEFEFKFK)	Self-assemble peptides	*Pseudomonas aeruginosa*		Tang et al., 2014
Synthetic peptides (CM3)	Liposomes			Lange et al., 2001
Melanoma antigen (MART)	PLGA nanoparticles		Immunomodulatory	Ma et al., 2012
Angiogenic peptide (AG-30)	Gelatin microspheres		Therapeutic and immunomodulatory	Nishikawa et al., 2009
Synthetic peptides (SIINFEKL)	PLGA nanoparticles			Sneh-Edri et al., 2011


#### Cyclosporin A

This is a non-ribosomal, deca-peptide which is cyclic and comprises hydrophobic amino acid residues. This peptide has been isolated from the fungus *Tolypocladium inflatum* and well-studied. Cyclosporin A has been used to treat nephrotic syndrome, refractory Crohn′s disease, ulcerative colitis, aplastic anemia, myasthenia gravis, dermatomyosistis, biliary cirrhosis, psoriasis, and severe cases of rheumatoid arthritis, and it prevents transplanted organ rejection, acting as an immunosuppressor (Reis et al., 2006b; Kumari et al., 2010). The peptide inhibits T cell proliferation by interfering with enzyme activity by a calcium-dependent serine threonine phosphatase known as calcineurin. The enzyme dephosphorylates the activating transcription factor (NFAT) that is responsible for stimulating Interleukin (IL)-2 expression, which is directly related to T cell proliferation (Reis et al., 2006b). However, cyclosporin A presents side effects including cytotoxicity, nephrotoxicity, neurotoxicity and hepatotoxicity (De Mattos et al., 2000). The peptide is lipophilic and can easily be encapsulated into nano delivery systems (Reis et al., 2006a). Thus, this peptide was encapsulated into different delivery systems in order to enhance its bioavailability and reduce the aforementioned collateral effects (Shi et al., 2013; Romero et al., 2016).

Cyclosporin A has been encapsulated in amorphous nanoparticles, evaluated for enhanced dermal bioavailability in an *ex vivo* setting. Using a pig ear skin model, strips of skin were exposed to a hydrogel with 5% hydroxylpropylcellulose and either 5% cyclosporin A nanoparticles or free cyclosporin A. It was found that the cyclosporin A nanoparticles significantly increased its penetration, by about 6 times compared to free peptide (Romero et al., 2016). These results would recommend the use of this nano delivery system for topical administration. Moreover, in order to manage extra ocular disorders such as keratoconjunctivitis sicca, chitosan nanoparticles loaded with cyclosporin A have been investigated. This nanoformulation permits the corneal and conjunctival surface to have contact with prolonged exposure to this peptide without affecting the inner ocular structures, as well as avoiding systemic exposure (De Campos et al., 2004). Commercially, Sandimmune^®^ and Neoral^®^ are micro-emulsion based formulations of cyclosporin with the ability to self-emulsify, and they possess improved bioavailability and pharmacokinetics. Available as capsules, oral solution and injection, they are administered to overcome transplant organ rejection (Curtis et al., 1998; Martins et al., 2007). Moreover, to improve the bioavailability of cyclosporin A, other chitosan nanoparticles loaded with peptide have demonstrated better pharmacokinetics (1.8 fold increase in area under concentration against time (AUC) when compared to the commercial micro emulsion Neoral^®^ (El-Shabouri, 2002).

Furthermore, in a clinical setting Shi et al. (2013) studied a cyclosporin A encapsulated into a co polymer of polylactic-co-glycolide-co-caprolactone nanoparticles. A clinical study with high-risk keratoplasty patients implanted cyclosporin A nanoparticles in the inferior anterior eye chamber post surgery, and patients were observed over a year (Shi et al., 2013). The transplantation was successful in 88% of the cases, with rejection suppression of the surgeries, and complete absorption of the implant was observed over the 6 months (Shi et al., 2013). The results indicated a new post-surgery transplant, which is biodegradable and effective over an extensive time period. Therefore, the formulation is a possible tool for prophylaxis to treat intraocular immune diseases. Overall, despite its innate hydrophobicity and cytotoxicity, such inherent drawbacks of cyclosporin A can be mitigated with an adequate drug delivery system.

#### Nisin

Nisin is a bacteriocin with unusual amino acids formed by enzyme -mediated post-translational modification (Cotter et al., 2005) and belongs to the lantabiotic class. This peptide contains dehydrated residues of serine and threonine, which result in Dehydroalanine (DHA) and Dehydrobutyrine (DHB) amino acids (Schnell et al., 1988). Further cysteine residues and unsaturated amino acids lead to the formation of lanthionine and B methyllanthionine residues, which in turn form rings in mature bacteriocin due to intermolecular cross linking (Bierbaum and Sahl, 2009). Additionally, nisin is a polypeptide with 34 amino acids produced by *Lactococcus lactis*. There are two variants of nisin (A and Z); the A variant has histidine at position 27 while Z has asparagine at that position. The difference in structure is advantageous to nisin Z as it has a solubility and ability to diffuse which is crucial for food (Mulders et al., 1991). Nisin has a broad spectrum against Gram-positive bacteria and is generally non-cytotoxic against mammals (Maher and McClean, 2006). Hence, it is useful in extending food shelf life against contaminants. Below are some cases where nisin was loaded into a nano delivery system in order to improve its bioavailability.

This AMP has been encapsulated in liposomes to study its activity in milk fermentation. Importantly, it was shown that, when encapsulated, nisin Z did not disturb cheddar cheese fermentation. The liposomes were stable, releasing the peptide for 27 days at 4°C (Laridi et al., 2003). The encapsulated peptides were released most highly in milk, followed by phosphate buffer saline and whey. This demonstrated the stability of nisin loaded liposomes in milk, a medium that is used to ferment cheddar cheese (Laridi et al., 2003). Thus, with stable and prolonged release of peptides, these nano-carriers provide an alternative food preservative for cheese production that could offer extended shelf life and quality.

In addition, nisin has been encapsulated into solid lipid nanoparticles (SLP) (Prombutara et al., 2012), aiming to be used as a preservative against food contaminants. Nisin encapsulated into SLP released the peptide over 25 days, compared to the free nisin from a dialysis bag during a drug release study observed for 3 days. Then, nisin in either free or encapsulated form was evaluated for anti-microbial activity against two strains: *Listeria monocytogenes* DMST 2871, for 20 days compared to 1 day for the free peptide, while *Lactobacillus plantarum* TISTR 850 was inhibited for 15 days, compared to 3 days when exposed to free nisin (Prombutara et al., 2012). This study demonstrated the advantage of encapsulating nisin into SLP.

From the food industry application to an alternative antifungal therapeutic, this peptide has been encapsulated into polymeric nanoparticles comprising polycaprolactone. For topical administration of nisin against either acute or chronic candidiasis, nanoparticles were prepared, characterized and then studied *in vitro*, where permeation experiments were performed by Franz diffusion cells using pig vaginal mucosa (Coli et al., 2016). Encapsulated nisin showed a similar antimicrobial activity against *Candida albicans* strains as free nisin, which indicated the potential of encapsulated nisin as a prophylactic against Candidiasis and other gynecological infections. Additionally, the encapsulated nisin had permeation potential as well as gradually being released in the pig vaginal mucosa following permeation studies (Coli et al., 2016).

Further, nisin has been tested against *Staphylococcus aureus* when encapsulated in nanofibers made up of polyvinyl alcohol, wheat gluten and zirconia (Wang et al., 2015). These nanofibers presented a gradual release of nisin after 7 h and *Staphylococcus aureus* inhibition (Wang et al., 2015). All these results demonstrated that nanofibers containing nisin might be useful for wound dressing, as well as for active food packaging material.

#### Vancomycin

Vancomycin is a glycopeptide used in treating multi-resistant Gram-positive bacteria such as Staphylococci, Enterococci, Clostiridia, and *Diphterois bacilli*. Vancomycin hydrochloride is among the first-generation glycopeptide antibiotics known to interfere in the poiesis of phospholipids and in preventing both bacterial cell wall formation and reproduction (Reynolds, 1989; Yang et al., 2012). It binds with the peptide region of terminal D-alanine-D-alanyl of a peptidoglycan precursor. This peptide is the last line of defense against *b*-lactam resistant bacteria and for patients allergic to penicillin (Cheung and Diprio, 1986). However, the administration of this peptide presents collateral effects. These includes ototoxicity, neuromuscular blockade and renal toxicity (Kinik and Karaduman, 2008). Therefore, in order to minimize these side effects, this peptide has been encapsulated in nano delivery systems and extensively studied (Looss et al., 2001; Gavini et al., 2004; Yousry et al., 2017). For instance, vancomycin has been encapsulated in polymeric nanoparticles either made up of Eudragit or PLGA and a combination of both polymers. These vancomycin-loaded nanoparticles were tested *in vivo* against albino rabbits through a Draize test, which proved to be non-irritating and safe for ophthalmic administration. Furthermore, microbiological susceptibility tests were performed and it was reported that peptide encapsulated nanoparticles remained at a higher concentration and for a longer time in the body compared to the free form. Hence, fewer doses of treatment will be required for nanoformulation (Yousry et al., 2017).

Additionally, vancomycin has been loaded into biodegradable polycaprolactone (PCL) microparticles. This study investigated microparticles loaded with vancomycin that were prepared by solvent evaporation extraction, followed by co administration of these microparticles with injectable bone substitute synthetic calcium phosphate, evaluating their ability to prevent infection post-surgery (Looss et al., 2001). These microparticles were uniform in size, spherical, showed high encapsulation efficiency, were cyto-compatible and prolonged peptide release by 26% in a week (Gavini et al., 2004). A prolonged release is advantageous in that vancomycin will continue preventing *Staphylococcus* growth over time, and there will be no need for a patient to take antibiotics post-surgery. Thus, this nanoformulation presents an alternative therapy (Looss et al., 2001). Lastly, liposomes loaded with vancomycin and chitosan coated have been investigated and presented improved pharmacokinetics, when compared to either free form or peptide-loaded liposomes without the covering *in vivo*. Chitosan-coated liposomes with the peptide had low concentration in the kidney and high concentration in the lungs, compared to free vancomycin and vancomycin-loaded liposomes. In this way, it is possible to reduce nephrotoxicity caused by vancomycin, while increasing the effect on lung infection, which is significant in a clinical setting (Yang et al., 2015). Therefore, the presence of chitosan in coated liposomes was important, in preventing a burst of vancomycin release and hence allowing prolonged systemic circulation.

#### Polymyxin

Polymyxin is a peptide isolated from *Bacillus polymix* which inhibits a broad range of Gram negative bacteria (Kumarasamy et al., 2010; Velkov et al., 2013). Additionally, it is effective against resistant Gram negative strains. Its mechanism of action is via attachment to acidic phospholipids and lipopolysaccharide bacterial cell membrane, causing the leakage of intracellular components, ending in death (Brogden, 2005; Arnold et al., 2007). Polymyxin B is widely used for urinary tract infection, meningitis, mucocitis, otitis, periodontitis and lung, ear, ocular and wound infection treatment (Arnold et al., 2007; Henao and Ghaffari, 2016; Myint et al., 2016; Rigatto et al., 2016). However, adverse side effects have been reported for polymyxin, such as neurotoxicity and nephorotoxicity, as well as a risk of promoting skin pigmentation (Gallardo-Godoy et al., 2016).

In order to overcome bioavailability and decrease the side effects of polymyxin, this peptide was also nanoformulated. The antimicrobial activity of polymyxin was investigated through its encapsulation into SLP *in vitro*. The SLP were prepared by double water in oil in water emulsion technique and characterized, followed by antimicrobial tests. These SLP were effective in inhibiting total growth of resistant *Pseudomonas aeruginosa* in comparison to the peptide alone over a variation of strains. The peptide showed its activity at 10 mg/mL, while polymyxin loaded nanoparticles were active at a much lower concentration, thus presenting themselves as a novel antibiotic-drug therapy (Severino et al., 2017).

Moreover, these AMPs have been encapsulated into liposomes and evaluated against resistant bacterial strains. These resistant strains are known to modify their cell surface, to thus reduce their interaction with the antibiotic through a complex phenomenon caused by adaptive or mutational response. Polymyxin loaded liposomes demonstrated a high cellular uptake when examined through immunocytochemistry studies (Alipour et al., 2008). The liposome-entrapped polymyxin (16 μg/mL) showed a higher uptake compared to free polymyxin (64 μg/mL). This result affirmed that when the peptide is loaded in a nano-carrier it presents a high penetration to clinical isolates of *Pseudomonas aeruginosa* (Alipour et al., 2008). In this way, it was demonstrated that liposomal polymyxin overcame bacterial surface modification and permeability, which is an additional quality sought when implementing nano delivery systems. When the peptide was incorporated into liposomes, it showed lower values of minimum inhibitory concentrations (MICs), when compared to its free form against *Pseudomonas aeruginosa*. Furthermore, immunocytochemistry and microscopy studies showed a high penetration of encapsulated polymyxin B in resistant *Pseudomonas aeruginosa* compared to its free form (Alipour et al., 2008). Therefore, nano-carriers have been implemented to encapsulate polymyxin, presenting positive results.

#### Supplementary Antimicrobial Peptides Involved in Passive Nano Delivery Systems

Apart from the above listed AMPs, there is an ever growing body of research involving natural or synthetic peptides. Such studies have used nano-carriers to encapsulate AMPs as therapeutics and immunomodulators (Leite et al., 2017). Small synthetic peptides have been evaluated against resistant bacteria *in vitro;* these peptides are developed from computer algorithms which evaluate their possible interaction with the surface chemistry of known microorganisms (Cruz et al., 2017). For example, a synthetic peptide, GIBIM-P5S9K (ATKKCGLFKILKGVGKI), was encapsulated in PLGA and PLA nanoparticles and evaluated against bacteria *in vitro*. The study found that the encapsulated GIBIM-P5S9K inhibited growth of *Escherichia coli* 0157:H7, methicillin-resitant *Staphylococcus aureus* (MRSA) and *Pseudomaonas aeruginosa* strains at 10% the concentration of the free peptide, and these nanoparticles did not cause hemolysis (Cruz et al., 2017).

Further, some peptides with their innate ability to self-assemble have been explored as nano-carriers. For instance, Tang and colleagues utilized octa-peptides with a self-assembly capability. Synthetic peptides with amino acid sequence FEFEFKFK are rich in antiparallel β-sheet fibers and known to form a hydrogel. Using a Visking membrane which mimics the mucosa surface, this work showed separate hydrogels carrying model drugs, either lidocaine or flurbiprofen, to be effective as a local mucosa delivery gel, capable of carrying an additional drug when tested *in vitro*, as well as, an adhesive agent (Tang et al., 2014). The stiffer hydrogel (40 mg/mL) had higher retention rates of the drug compared to the less viscous hydrogel (20 mg/mL) when the release profile of the both hydrogels was evaluated (Tang et al., 2014). The peptide was used to host the model drugs, which provides a combinatorial therapy alternative, provided the incorporated drug has synergistic or additive efficacy.

Also as nano-carriers, amphiphilic peptides have been prepared and characterized by Zha et al. (2013) as a potential anti-cancer therapy. These peptides comprise a small peptide sequence (KLAKLAK)_2_ and self-assemble into nano-fibers when coupled with hyaluronic acid to form a complex (Zha et al., 2013). The peptide sequence is known to induce cell death in breast cancer cells over non-cancer epithelial cells (Standley et al., 2010). After physico-chemical characterization of the nano-fibers, the study demonstrated that the nano-fibers were cytoxotic against breast cancer cells *in vitro* in a gradual form which was associated with the degradation of the materials upon exposure to the hyalunodase enzyme (Zha et al., 2013). Thus, the nano-system could also incorporate a different drug to provide a combinatorial therapy.

Antimicrobial peptides as immunomodulators were explored by Ma et al. (2012) while studying the immune response of human dendritic cells. The study used PLGA nanoparticles, encapsulating melanoma antigen recognized by T cells (MART), prepared by emulsification followed by evaporation of oil in water emulsion. Then, the peptides either in their free or encapsulated form were exposed to matured dendritic cells *in vitro*. It was successfully demonstrated that the peptide loaded nanoparticles elicited a more effective immune response in comparison to the peptide alone (Ma et al., 2012).

In addition, as both therapeutic and immunomodulator, in order to develop a potential alternative treatment to Ischemic diseases, an angiogenic peptide (AG-30) was screened *in silico*, then encapsulated in microspheres and investigated *in vitro* and *in vivo*. This peptide had both antimicrobial and pro inflammatory activities (Nishikawa et al., 2009). The angiogenic peptide is an α helical peptide with a high percentage of hydrophobic residues that are cloned from a human cDNA library. With human aortic endothelial cells, AG-30 upregulated angiogenesis-related cytokines and growth factors *in vitro*. Moreover, AG-30 encapsulated in microspheres made up of gelatin increased the angiogenic score of ischemic-induced C57BI/6J mice hind limb (Nishikawa et al., 2009). The mice injected with AG-30 microspheres demonstrated more rapid blood flow recovery compared to those without the microspheres.

Regarding the application of nano delivery systems as a potential anti-tumor therapeutic, such systems can take advantage of the tumor microenvironment, which is over supplied with blood vessels but tends to lack lymphatic drainage (Narang and Varia, 2011). Nanoparticles can be retained in the tumor through what is known as the enhanced permeation and retention (EPR) effect (Narang and Varia, 2011; Leite et al., 2017). This effect can be explored via passive targeting, as the size of nanoparticles is key in enhancing retention.

Thus, nano delivery systems have been deployed to encapsulate a wide range of synthetic and natural AMPs, presenting valuable results, as has been highlighted by the case studies cited above.

### Antimicrobial Peptides in Targeted Nano Delivery Systems

**FIGURE 2:**
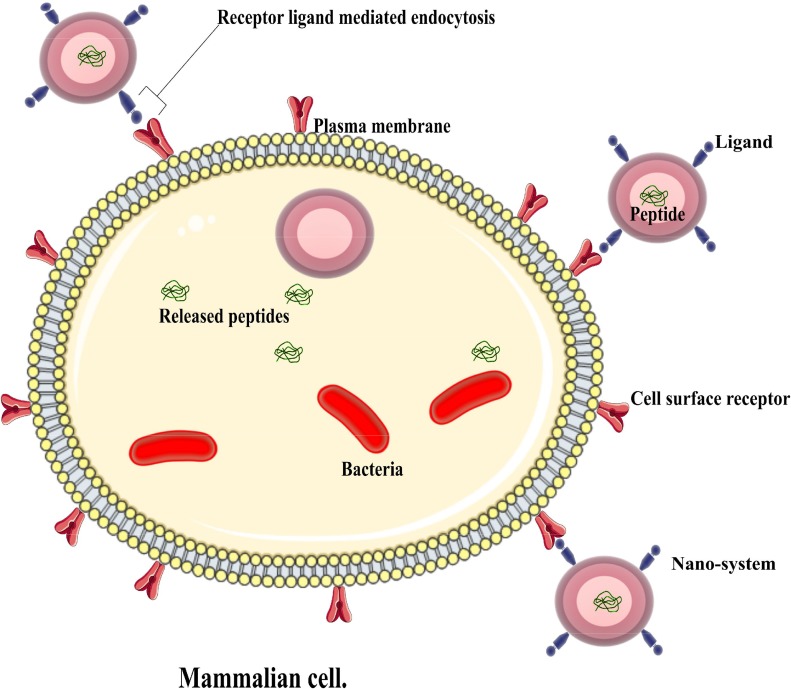
An illustration of an active nano system demonstrating antimicrobial peptide delivery into an infected cell.

For a more precise delivery of peptides of interest, nanosystems have been decorated or modified with other moieties to improve their delivery into intended sites (Liu et al., 2018) as depicted in **Figure 2**. Various approaches have been implemented over the years, and some drug delivery systems have employed AMPs as ligands to deliver other peptides or conventional drugs, while others have used other chemicals to help deliver AMPs (Solaro et al., 2010) as highlighted in **Table 2**. This system aids in circumventing natural barriers in organisms. Such barriers include the presence of low pH and enzymes in the gastrointestinal tract and mucosa and epithelial layers in the intestine (Harloff-Helleberg et al., 2017; Liu et al., 2018), which prevents the desired release of peptides to the intended site. The following section will examine such cases that have applied targeted delivery systems.

**Table 2:** An overview of antimicrobial peptides contained/adsorbed in various active nano delivery systems and their various biological applications.

Encapsulant	Nano delivery system	Ligand	Antimicrobial Target	Application	Reference
Vancomycin	Liposomes	Chitosan		Biodistribution	Yang et al., 2015
	Nanomicelles	Poly-ethylene Glycol			Brandenburg et al., 2012
	Alginate microparticles	Chitosan	*Pseudomonas aeruginosa*	Lymphatic system targeting	Coppi et al., 2006
Connexin 43	Albumin nanoparticle	Hyaluronic acid		Anti-retinal inflammation	Huang et al., 2017
Doxorubicin	PLGA nanoparticles	Candoxin (CDX)		Anti-tumor	Chai et al., 2017
Simvastatin	PLGA nanoparticles	mZD7349 peptide		Atherosclerosis treatment	Imanparast et al., 2017
Docetaxel	Chitosan nanoparticles	LFC131 peptide		Lung cancer	Wang et al., 2015
Epirubicin	PLGA nanoparticles	LFC131 peptide		Liver cancer	Di-Wen et al., 2016


Sneh-Edri et al. (2011) worked with a synthetic peptide in a targeted delivery system using polymeric nanoparticles. Their study investigated uptake and intracellular targeted drug delivery using two lines of mouse dendritic cells, namely DC 2.4 and B3Z hybridoma T cells, which were capable of recognizing the peptide sequence of interest (Sneh-Edri et al., 2011). It has been shown *in vitro* that PLGA nanoparticles loaded with a peptide sequence (SIINFEKL) and fluorescent marker (BSA-FITC) surface conjugated with an antigenic peptide residue affected intracellular trafficking, increased the degree of endocytosis and also induced a prolonged cross presentation of the decorated peptide surface (Sneh-Edri et al., 2011). This elevated uptake was directly reported based on the peptide linking to receptors of T cells, facilitating the delivery of the marker into the cells, which is an interesting approach for site-specific treatment.

In another study, vancomycin was carried in a modified fusogenic liposome. The peptide is known not to be active against gram negative bacteria due to its failure to cross the bacterial cell membranes (Nicolosi et al., 2010). Vancomycin was encapsulated into small unilamellar liposomes covered with phospholipid-cholestral hemisuccinate, which has an affinity to the eukaryote cell membrane, and then was tested for the antimicrobial activity against clinical isolates of *Escherichia coli* and *Acinetobacter baumanni*. Neither activity was observed for free vancomycin nor for the drug encapsulated in non-fusogenic liposomes, while the fusogenic liposome presented a minimum inhibitory concentration of 6 mg⋅ml^-1^ (Nicolosi et al., 2010). Therefore, surface modification of liposomes improved the performance of vancomycin.

In a different study, human serum albumin nanoparticles loaded with connexin 43 and coated with hyaluronic acid were utilized (Huang et al., 2017). This peptide is 43 kDa and an important isoform of transmembrane protein connexins. Cx43 plays a role in maintaining retinal homeostasis and possesses a potential to reduce edema, blocking the pathological opening of gap junction hemi channels from vascular leakage caused by retinal injury, as well as inflammation and neuronal death (Fraysse-Ailhas et al., 2007). *In vitro* CD44 positive-expressing human RPE cells (ARPE-19) presented higher cellular uptake of the peptide of hyaluronic acid coated albumin nanoparticles, compared to non-coated ones, as expected from the interaction of HA with CD44 receptors. Moreover, e*x vivo* fresh bovine eyes presented enhanced neural retinal and RPE/choroid penetration of the coated nanoparticles compared to the uncoated ones, which is consistent with the *in vitro* studies. Both nano delivery systems demonstrated slow peptide release up to 4 months, although the surface modified albumin nanoparticle showed improved delivery of connexin 43 mimetic peptide (Cx43) to the retina in the *ex vivo* experiments (Huang et al., 2017). Therefore, these systems present a more efficient therapy for the treatment of retinal inflammatory related diseases.

Additionally, AMPs may be adsorbed on the nano-carrier’s surface to facilitate the delivery of the drug of interest. For instance, in order to deliver an anticancer agent, doxorubicin, to the brain, red blood cell membrane-coated PLGA nanoparticles (RBCNP), which were also conjugated to a neurotoxin derived peptide, CDX, have been investigated. CDX is a peptide derived from candoxin, with high binding affinity to nicotinic acetylcholine receptors found on the brain surface expressed by endothelial cells. This peptide was coated on the surface of a polymeric PLGA nanoparticle, which was also coated with a red blood cell membrane to aid the passage across the blood brain barrier. The nanoparticles showed promising efficiency in delivering doxorubicin *in vitro*, with primary brain capillary cells isolated from Wistar rats and U87 human brain cells. Furthermore, an *in vivo* experiment took place using a glioma mouse model: nude mice were inoculated with tumor cells and treated with either free doxorubicin, doxorubicin loaded RBCNPs or doxorubicin loaded RBCNPs with CDX, and the survival curves showed a longer survival of the CDX group (28 days) in comparison with the other two, which survived 22.5 and 23.5 days, respectively. This result was further confirmed with glioma brain sections, showing that the CDX group induced more apoptosis when compared to the others (Chai et al., 2017). Thus, active targeted nanoparticles were better delivery systems than non-conjugated nanoparticles.

Moreover, a different study evaluated endothelial dysfunction recovery, aiming to improve atherosclerosis treatment. PLGA nanoparticles loaded with simvastatin were conjugated with a synthetic peptide mZD7349 and investigated *in vitro* against vascular cell adhesion molecule 1 (VCAM-1) expressed by activated human umbilical cord vascular endothelial cells (HUVECs) (Imanparast et al., 2017). Quantification of endothelial nitric oxide synthase, an enzyme responsible for repairing the vascular endothelium, showed that PLGA nanoparticles loaded with simvastin and conjugated to mZD7349 peptide expressed more of this enzyme compared to those non-conjugated nanoparticles with simvastatin (Imanparast et al., 2017). Due to active targeted delivery, more of the drug was presented in the vascular endothelium in this work.

Apart from targeting microorganisms, AMPs have been studied against cancerous tumors. Taking advantage of the structural alteration of most cancer cells, drug delivery systems have been implemented. Such structural changes include the plasma membrane exposure of the anionic phospholipid phosphatidylserine to the outer membrane, from its normal inner membrane position (Iwasaki et al., 2009). This in turn increases the membrane negative charge and transmembrane potential (Iwasaki et al., 2009; Teixeira et al., 2012). Cancer cells also express other biomolecules which can be targeted, such as chaperone proteins HSP90 and GRP78, sialic acid (Liu et al., 2018), heparin sulfate (Lee et al., 2015) and *O*-glycosylated mucines (Teixeira et al., 2012). Below, such cases will be examined.

Investigating an alternative therapy for lung cancer, Wang et al. (2015) used a peptide conjugate on chitosan nanoparticles loaded with docetaxel, a known anti-cancer drug, *in vitro*. The peptide LFC131 is an antagonist to a seven transmembrane G protein coupled receptor (CXCR4) (Dewan et al., 2006). The receptor plays a role in cell migration and metastasis of cancer and is overexpressed in lung, breast and prostate cancer (Tang et al., 2008). The antitumor effect of targeted and non-targeted nanoparticles, and free docetaxel against A549 lung cancer cells, was investigated *in vitro.* LFC131 peptide conjugated docetaxel-loaded chitosan nanoparticles presented enhanced cancer cell death, and greater caspase-3 activity compared to non-targeted and free docetaxel (Wang et al., 2015). Thus, the presence of an AMP enhanced receptor mediated interaction with cancer cells.

Di-Wen et al. (2016) encapsulated epirubicin in polymeric nanoparticles and conjugated them to LFC 131 peptide on its surface. They developed a liver-targeted drug delivery system and investigated anti-tumor activity *in vitro* and *in vivo* (Di-Wen et al., 2016). A linear type of FC131 peptide (D-Tyr-Arg-Arg-2-Nal-Gly) was used to target C-X-C receptor type 4 (CXCR4) that is overexpressed by hepatocellular carcinoma. *In vitro* with human hepatic carcinoma cells (HepG2), the study found that the targeted nanoparticles had a threefold uptake compared to the uncoated. Further, *in vivo* a biodistribution of FC131 peptide-targeted epirubicin-loaded PLGA nanoparticles was compared to uncoated, showing a higher concentration of the drug in the liver even after 24 h (Di-Wen et al., 2016), demonstrating the effectiveness of targeted delivery.

### Challenges and Limitations of Antimicrobial Peptides and Nanotechnology

At the present there are more than a dozen FDA approved peptide based drugs, which are not encapsulated into nano-carriers in the market. Such drugs include Prialt^®^ for chronic pain approved in 2004, Firmagon^®^ against prostate cancer (2008), Victrelis^®^ for Hepatitis C (2011), Victoza^®^ for Type II diabetes mellitus (2013), Kyprolis^®^ for multiple myeloma (2012), Lyxumia^®^ for Type II diabetes approved in 2013 (Graul et al., 2013). With exception of Omontys^®^ for anemia which was approved in 2012, and later withdrawn from the market due to hypersensitivity within a year (Kaspar and Reichert, 2013), most peptide based drug are available in the market. Some successful peptide based drugs include Zoladex^®^ and Copaxone^®^, which have sold more than 5.2 billion US dollars between them (Habib-Valdhorn, 2013). Intriguingly, there are no FDA approved synthetic AMPs in the market (Molchanova et al., 2017). Emphasizing on cost effectiveness, the production of a synthetic peptide alone is estimated between US $300–500 per gram (Bray, 2003; da Costa et al., 2015). Bray estimated production cost for a 5000 Da molecular mass peptide is 100 fold the production for 500 Da molecular mass of small molecules, instead of the expected 10 fold difference (Bray, 2003), which overall limits investment of producing alternative therapeutics. Furthermore, depending upon the drug delivery system selected, incorporating the peptides may contribute additional production cost.

The fact that most drug delivery system that carry peptide are still under research, there is a public concern of the peptides safety when compared to small molecule drugs (Otvos and Wade, 2014). Peptide based drug suffer from proteolytic instability *in vivo*, among other shortcomings (Otvos and Wade, 2014). For instance Tyrothricin and Bactericin (combination of polymyxin B and neomycin) causes hemolysis and nephrotoxicity respectively, and are both administered as topical agents (Molchanova et al., 2017). Currently there is a number of AMPs implemented in clinical trials that have presented side effects and subsequently removed from the shelf. The AMPs that failed in clinical tests include NVB-302 (phase I), POL7080 (phase II), Iseganan, Omiganan, Surotomycin both at phase III (Kosikowska and Lesner, 2016; Greber and Dawgul, 2017; Sierra et al., 2017). This has drawn the efforts of the pharmaceutical industry, toward investing on AMPs for topical use rather than parenteral application, and this limitation may be overcame by nanotechnology.

In general, the methods of preparing nano delivery systems present some drawbacks, such as peptide exposure to organic solvent, shear stress caused by either sonication or mechanical homogenization, which in turn could change the morphology of a peptide and its activity (Mohammadi-Samani and Taghipour, 2014). The encapsulated peptide might also interact with walls of nano-carriers in the nano-environment, and its release might be affected, in a phenomenon called incomplete release (Mohammadi-Samani and Taghipour, 2014). Stability and aggregation are another hindrance for nano-system preparation, as when encapsulated into a hydrophobic system containing an aqueous layer some hydrophilic peptides may leak from the systems (Mohammadi-Samani and Taghipour, 2014). This results to aggregation of the nano-carriers. Additionally, the most pressing challenge of deploying nano-carriers is the cost effectiveness. The product Nutropin Depot^®^, which is PLGA loaded with human growth hormone, was introduced on the market in 1999 by Alkernes and Genetech companies but later was discontinued due to cost-effectiveness (Stevanovic and Uskokovic, 2009).

Other nano delivery systems require the use of high temperature or very low temperature, which may denature the peptides as one of the methods of producing SLP (Gallarate et al., 2011). Others nano-carriers as polymeric nanoparticles require use of organic solvent which may be toxic and influence the structure of synthetic peptide upon its encapsulation all these add to the hurdles of AMPs and nanotechnology. Additionally, achieving optimum drug loading, identification of right polymer with an acceptable safety profile, maintaining the peptide integrity during processing as well as storage while maintaining its stability and bioactivity (Wang et al., 2002) are other important hurdles.

The “holy grail” of AMPs is developing them for oral delivery which is patient friendly and non-invasive (Kumar et al., 2006). Currently, some peptides such as ramoplanin, surotomycin, and one of the failed AMPs NVB-302 are being developed for treatment of infections caused by *Clostridium difficile* (Molchanova et al., 2017). Production of such drugs is still an ongoing challenge given the number of obstacles above stated.

## Conclusion

Even though there are still challenges regarding these novel AMPs incorporated in drug delivery systems, such as low efficiency of encapsulation, finding a suitable delivery system for the peptides, and inherent complex properties of peptides, a great deal of work is being carried out to optimize such nano-systems carrying AMPs. Currently, there are a few clinical trials that are implementing AMPs loaded in nano-carriers, even though there are a relatively higher number of basic research studies which do not go further than *in vitro* experiments. There are many opportunities in the near future, as AMPs are becoming more widely and thoroughly researched. Therefore, further investigation is required to set up adequate *in vivo* models, because more physiological barriers and immunological responses are involved with animals upon exposure to AMPs, and most are not yet well understood. The complexity is amplified in clinical trials, due to patient incompliance and individual dose response to AMPs. These are some of the challenges for the application of nano-carriers to deliver AMPs. Nonetheless, investing in natural peptides and nano delivery systems developed from natural polymers might be the way forward in the foreseeable future.

## Author Contributions

LB was partially involved in writing the introduction and, subsequently, the remaining manuscript. MC wrote the introduction. TR, SD, and OF revised the manuscript.

## Conflict of Interest Statement

The authors declare that the research was conducted in the absence of any commercial or financial relationships that could be construed as a potential conflict of interest. The reviewer SD and handling Editor declared their shared affiliation.
